# Unpacking Brand Fandom: Triadic Brand-Related Perceptions and Fan Behaviors

**DOI:** 10.3390/bs16091529

**Published:** 2026-08-30

**Authors:** Yonju Lee, Boyoung Kim

**Affiliations:** Seoul Business School, aSSIST University, Seoul 03767, Republic of Korea; yonju.lee@stud.assist.ac.kr

**Keywords:** brand fandom, brand innovativeness, self-transcendent emotion, self-brand connection, perceived product value, need for self-expression

## Abstract

This study examines the influence of consumers’ brand-related perceptions on brand fandom behaviors. In particular, the study focuses on how brand experience, consumers’ perceived product value, and perceived brand innovativeness affect sharing and advocacy behaviors as well as creation behaviors, mediated by self-transcendent emotion and self-brand connection. This study also investigated the moderating role of the need for self-expression in these mediating pathways. To test the proposed hypotheses in the context of high-tech brands, structural equation modeling was employed, and data were collected from 635 Tesla owners and prospective buyers in the United States and Republic of Korea. All ten hypothesized paths were significant. The two mediators, however, correlated only weakly with each other while each correlated strongly with the behaviors, indicating two largely independent routes to the same fan behaviors rather than two indicators of a single bond. The need for self-expression strengthened all four second-stage paths, so brand fandom is best described as a two-stage process with two distinct routes and a shared expressive condition. The findings suggest that designing for only one of the two routes reaches only part of a brand’s potential fan base.

## 1. Introduction

Today, consumers watch real-time announcements of new products from brands they are attached to and voluntarily create unboxing videos. Furthermore, they gather in digital communities to collectively interpret a brand’s future vision and even step forward as voluntary advocates when a company faces adverse publicity. This kind of active fan behavior is no longer a phenomenon limited to a select few passionate consumers. According to a recent industry survey, approximately 73% of U.S. adults identified as “superfans” of at least one brand, and brand fandom is being observed across various industries, including technology, automotive, entertainment, and media (National Research Group, 2023). Such consumers gather in brand communities documented across product categories (Muñiz & O’Guinn, 2001), and the practices through which they engage with the brand and with one another create value for the brand itself (Schau et al., 2009). The present study aims to examine this process in the context of high-technology brands.

Consumers experience brands sensorially and intellectually through various touchpoints, such as stores, advertisements, and digital interfaces (Brakus et al., 2009). By using products directly or encountering them indirectly through other users’ reviews, consumers perceive both the product’s quality and the social image it conveys to them (Sweeney & Soutar, 2001). Furthermore, consumers are exposed not only to a brand’s technical excellence but also to innovation cues such as the company’s creativity, spirit of challenge, technological leadership, and vision for the future (Shams et al., 2015). By synthesizing these multi-layered perceptions, consumers form an overall impression of the brand and maintain their relationship with the brand.

Consumers perceive brands they are attached to as symbolic objects that express their identity (Escalas & Bettman, 2005). By resonating with the product experience, technological achievements, and future vision conveyed by a brand, consumers may also perceive that brand as something special and awe-inspiring (J. Kim et al., 2021). Additionally, it has been confirmed that brand content conveying emotional and experiential value is more effective at driving voluntary sharing and creative participation among loyal fans than promotional messages (Malodia et al., 2024).

Such identity connection and emotional engagement do not merely linger in consumers’ minds but play a crucial role in driving active behavior. According to prior research, consumers who align a brand with their self-identity are more likely to actively engage in voluntary advocacy and recommendations (Stokburger-Sauer et al., 2012), and intense emotional engagement with a brand leads to active participation, such as content creation (Dessart et al., 2015).

How brand perception comes to produce action of this kind has been approached in research on consumer–brand relationships through concepts such as satisfaction, loyalty, brand love, attachment, and identification (Fournier, 1998; Fullerton, 2005; Thomson et al., 2005; Carroll & Ahuvia, 2006; Batra et al., 2012; Stokburger-Sauer et al., 2012; Na et al., 2023; Damaschi et al., 2025). This body of work has been valuable in characterizing the state of the consumer–brand relationship, although the psychological route by which brand perception is translated into brand-directed action has received comparatively less attention. Where such a route is specified, it is often represented by a single mediating construct, and affective responses and identity-based responses have tended to be treated as closely related indicators of relationship strength. The two have in fact been examined in separate research streams, and they appear to differ in the direction of the relation they describe: self-transcendent emotion is oriented outward, toward the brand as an object, with the self correspondingly de-centered (Keltner & Haidt, 2003; Zickfeld et al., 2019), whereas self-brand connection describes the brand being drawn into the self-concept (Escalas & Bettman, 2005). Consumers who are frequently moved by a brand and consumers who define themselves through it may therefore not entirely overlap. A further consideration is that accounts of this kind often conclude at the internal response, which leaves relatively unexplored the conditions under which such a response comes to be expressed in observable behavior.

Building on these considerations, this study examines brand experience, perceived product value, and perceived brand innovativeness as antecedents of two parallel psychological routes—self-transcendent emotion and self-brand connection—leading to sharing and advocacy behaviors and to creation behaviors. Specifying the two routes in parallel allows their relative contributions and their degree of independence to be assessed, which a single-mediator specification does not permit. The study further considers the need for self-expression as a moderator of the second stage, in order to examine the conditions under which internal responses are translated into observable fandom behaviors. Ultimately, this study verifies within an integrated model that brand fandom forms through two distinct routes rather than a single bond of graded strength, and provides strategic implications for fan-base management that address both.

## 2. Literature Review and Hypotheses Development

### 2.1. Brand-Related Perceptual Antecedents

Brand-related perceptual antecedents refer to multi-layered perceptual responses that consumers form through direct and indirect contact with a brand (Cui et al., 2025; Klaus & Maklan, 2013). Consumers do not evaluate a brand solely based on the image presented by the company, product features, or value for money. While experiencing company-driven stimuli such as products and digital touchpoints, consumers also interpret the product’s intrinsic value and social significance through actual usage experiences, others’ evaluations, online reviews, community discussions, and user-generated content (Paramita et al., 2021; J. Xu et al., 2026). Furthermore, how a brand presents not only the products it currently offers, but also its future-oriented technologies, values, vision, and innovation trajectory serves as a crucial cue for consumers’ brand perception (Bu et al., 2023). From this perspective, brand experience, perceived product value, and brand innovativeness can be understood as three distinct brand-related perceptual antecedents, operating at the experiential, evaluative, and innovative/symbolic levels respectively, rather than as dimensions of a single higher-order construct. The three are distinguished by what the perception appraises: the consumer’s own response to the brand, the worth of what the product delivers, or the brand itself as a source of new ideas and future direction. Each may be formed from the consumer’s own encounters or from others’ accounts—the criterion concerns what is appraised, not where the information came from—and a consumer may hold one without holding the others. The same criterion indicates what lies outside the construct as it is used here. Brand image and brand personality are not included, since they characterize what a brand is like rather than appraise what it delivers or can deliver; nor are brand trust and brand attitude, which presuppose accumulated perception rather than constituting it and would stand between perception and response in the model.

First, brand experience is defined as the subjective, internal, and behavioral responses of consumers triggered by brand-related stimuli—such as brand design, identity, packaging, communication, and in-store environments—and consists of four dimensions: sensory, affective, intellectual, and behavioral (Schmitt, 1999; Brakus et al., 2009). Brand experience is formed not only during the product search, purchase, and usage processes but also occurs across various brand touchpoints—such as advertisements, websites, and events—and throughout the entire customer journey (Verhoef et al., 2009; Schmitt, 2015; Lemon & Verhoef, 2016). In particular, the digital environment has been discussed as a context that expands the scope of brand experience through images, videos, interactivity, information search, and participatory activities, thereby facilitating consumers’ cognitive, emotional, and behavioral activities related to the brand (Hollebeek et al., 2014; Paramita et al., 2021). Building on this foundation, brand experience can be conceptualized as a perceptual factor at the experiential level that consumers form through brand-related stimuli at various brand touchpoints throughout the customer journey (Schmitt et al., 2014).

Perceived product value is a perceptual dimension through which consumers evaluate the value provided by a brand in the context of purchase and use, based on the product’s quality, performance, utility, cost (Zeithaml, 1988), as well as its social significance (Sweeney & Soutar, 2001). Recent research on customer value emphasizes that, amid technological innovation, digital touchpoints, and the proliferation of co-creation and relationship marketing, consumers evaluate brands not based on image or communication, but through the utility and social significance they perceive during actual use (Zauner et al., 2015; Zeithaml et al., 2020; Blut et al., 2024). Consumers form these evaluations by using the product directly or by observing others’ experiences with it (Payne et al., 2017; Bickart & Schindler, 2001; Babić Rosario et al., 2016). Particularly in the context of product-based brand evaluation, quality and performance values demonstrate how faithfully a product fulfills the brand’s functional promises, while social value can be understood as a key dimension that explains the significance of product use in shaping consumers’ social self-concept and positive self-image (Sweeney & Soutar, 2001; Rintamäki & Saarijärvi, 2021).

Brand innovativeness refers to the extent to which consumers perceive a specific brand as new, creative, a leader in market change, and capable of continuous innovation. Henard and Dacin (2010) suggested that the perception of innovation can have a positive impact on consumer excitement, loyalty, and brand image. Shams et al. (2015) extended this concept to the brand level and conceptualized consumer-perceived brand innovativeness as a distinct perception of the brand as a whole, rather than as an evaluation limited to the innovativeness of individual products (Lowe & Alpert, 2015). In this context, brand innovativeness is not limited to technical or functional novelty but includes symbolic interpretations of the new ideas, creativity, distinctiveness, leadership, and future potential that the brand presents to the market. In particular, the innovativeness of high-tech brands leads consumers to interpret the brand not merely as a current product provider but as an entity that represents the future potential of the industry; innovativeness and excellence serve as factors that cause consumers to perceive the brand as something special and awe-inspiring (J. Kim et al., 2021). Therefore, brand innovativeness can be viewed as a symbolic perception of a brand’s creativity and future-oriented nature, serving as a crucial foundation for understanding strong consumer-brand relationships (Chen et al., 2024; Hubert et al., 2017).

#### 2.1.1. Brand-Related Perceptual Antecedents and Self-Transcendent Emotion

Self-transcendent emotion refers to an emotional state in which consumers are deeply moved when encountering a brand, respond with emotional warmth, and experience the brand as something special and inspiring (Zickfeld et al., 2019; J. Kim et al., 2021). In positive psychology, self-transcendent emotions have been treated as a distinct emotional category in which attention is directed toward an object beyond the self, transcending one’s immediate needs or desires (Stellar et al., 2017). “Being moved” and “awe,” representative emotions in this category, are both explained by a common emotional structure in which the consumer’s sense of self-salience is reduced, leading to an emotional elevation that transcends the self (Yaden et al., 2017; Abatista & Cova, 2023; Pizarro et al., 2021).

In this regard, Zickfeld et al. (2019) conceptualized the emotion that people describe with terms such as “moved,” “touched,” and “heartwarming” as “kama muta,” and demonstrated that this emotion can be experienced relatively universally across cultural contexts. Keltner and Haidt (2003) explained awe by identifying vastness and the need for cognitive accommodation as its core elements. This sense of awe is experienced not only in nature or art but also in consumer contexts; Cavanaugh (2025) discussed how brands can evoke awe in consumers through various activities, such as advertising, brand communication, and brand experiences (Yan et al., 2024). Thus, brand-related perceptual antecedents do not merely constitute simple cognitive evaluations but can also foster self-transcendent emotional responses in consumers, such as being moved, inspired, and awe.

First, an intense brand experience can extend beyond cognitive evaluation to become a transcendent customer experience that produces a deep emotional bond with the brand (Brakus et al., 2009; Schouten et al., 2007; Mostafa & Kasamani, 2021). What distinguishes such an experience appears to be the degree of absorption it demands: an experience that occupies more sensory and cognitive attention than the consumer anticipated displaces the ordinary frame of reference, which is the appraisal condition under which a self-transcendent response is held to arise (Keltner & Haidt, 2003). Immersive brand experiences evoke awe and, through immersion, strengthen brand salience and purchase intent (Lemon & Verhoef, 2016; Errmann, 2025).

Perceived product value can be linked to a sense of transcendence through a product’s qualitative excellence and social significance, and a comprehensive meta-analysis establishes perceived value as a determinant of consumer judgment and behavior (Blut et al., 2024). Craftsmanship, technical performance, and superior user experience lead consumers to perceive the product not merely as an object of functional utility but as one embodying excellence and uniqueness, and it is this perception of excellence that provides the basis for an emotional response extending beyond the self. Consumers experience awe during product encounters when product attributes evoke timelessness and require cognitive accommodation (Guo et al., 2018). Recent research has also suggested that product-related perceptions—such as environmental benefits, perceived enjoyment, and relative advantage—can enhance awe experiences and recommendation intentions among electric-vehicle consumers (Rahi et al., 2026).

Finally, innovative brands present novelty and a forward-looking vision that exceed consumers’ existing expectations, allowing them to experience the brand not merely as a provider of products but as a symbol of industry transformation and a vision for the future (Shams et al., 2015; Aaker, 2023). A reputation for product innovation heightens consumer excitement and positive response (Henard & Dacin, 2010). J. Kim et al. (2021), in developing brand awe as a construct, identified brand innovativeness as one of its principal antecedents. The reason lies in what innovativeness signals: a brand judged capable of what the category has not yet delivered cannot be assimilated into the consumer’s existing expectations of that category, and this novelty, perceived vastness, and consequent need for cognitive accommodation are the conditions under which awe and inspiration arise (Keltner, 2025; Hagtvedt, 2025). Based on the foregoing discussion, the following hypotheses were proposed.

**H1.**  *Brand experience has a positive (+) effect on self-transcendent emotion.*

**H2.**  *Perceived product value has a positive (+) effect on self-transcendent emotion.*

**H3.**  *Brand innovativeness has a positive (+) effect on self-transcendent emotion.*

#### 2.1.2. Brand-Related Perceptual Antecedents and Self-Brand Connection

Escalas (2004), in developing the self-brand connection concept, describes it as the incorporation of a brand into the consumer’s self-concept, in which brand meaning is used to construct and communicate self-identity. Consumers do not merely use brands as simple means of utility; rather, they utilize them as symbolic resources to express who they are and convey a specific image to others (Belk, 1988; Fournier, 1998; Chernev et al., 2011). In particular, when brand meaning is linked to a consumer’s self-image, reference group, and social significance, the brand can transform from a functional choice into an identity resource that constructs self-concept (Escalas & Bettman, 2005; Zogaj, 2024).

Brand-related perceptions provide the foundation for forming a self-brand connection. Emotional brand attachment strengthens when brand personality aligns with a consumer’s actual or ideal self (Malär et al., 2011), and brand–self similarity, brand distinctiveness, brand social benefits, brand warmth, and memorable brand experiences all influence consumer–brand identification (Stokburger-Sauer et al., 2012). What these have in common is that consumers evaluate a brand not only on functional attributes but on how well it aligns with their self-image, what social significance it offers, and how distinctive and memorable the experience is; perceptions of this kind contribute to accepting the brand as an object linked to self-identity (Bhattacharya & Sen, 2003).

Brand experience serves as a touchpoint through which consumers connect a brand to their self-concept, and memorable brand experiences are among the key antecedents of consumer-brand identification (Büyükdağ & Kitapci, 2021). Brand experience is not merely a matter of contact frequency; it is transformed into self-brand connection depending on how positively and self-consistently consumers interpret their experiences at each touchpoint (Zogaj, 2024). Through meaningful experiences with a brand, consumers come to view it not merely as an object of use but as an integral part of their lifestyle, values, and personal narrative (Vernuccio et al., 2015; Batra et al., 2012), and in the smartphone context, brand experience has a significant effect on self-brand connection (Mostafa & Kasamani, 2021).

Perceived product value contributes to self-brand connection through its quality/performance and social value dimensions (Sweeney & Soutar, 2001), which align with the process by which consumers integrate brand meaning into their self-concept. Furthermore, prior studies have shown that consumers’ perceived value can influence the formation of self-brand connection, and that this connection can lead to brand loyalty and positive consumer responses (Thomson et al., 2005; Lin et al., 2017; Yeh et al., 2016).

Brand innovativeness offers consumers more than product performance; it serves as a symbolic resource that allows them to project a self-image open to new technologies and future possibilities (Shams et al., 2015). Consumers who define themselves as innovative are more likely to associate themselves with innovation-oriented brands, and brand innovativeness is a key brand-management factor influencing brand commitment (Eisingerich & Rubera, 2010); a reputation for product innovation likewise strengthens positive responses and loyalty (Henard & Dacin, 2010). In light of the above, the following hypotheses were proposed.

**H4.**  *Brand experience has a positive (+) effect on self-brand connection.*

**H5.**  *Perceived product value has a positive (+) effect on self-brand connection.*

**H6.**  *Brand innovativeness has a positive (+) effect on self-brand connection.*

### 2.2. Brand Fandom Behaviors

Brand fandom goes beyond brand preference or repeat purchases to encompass emotional attachment mediated by the brand (Thomson et al., 2005), the integration of self-identity and the brand (Escalas & Bettman, 2005; Stokburger-Sauer et al., 2012), a sense of community belonging (Muñiz & O’Guinn, 2001), and the active co-creation of brand meaning and value (Laroche et al., 2012; Swaminathan et al., 2020), constituting a relational and collective consumption phenomenon (Fuschillo, 2020; Jahn & Kunz, 2012; Preiksaitis & Pearson, 2025).

Fandoms can form not only around media content but also around products, brands, and consumption philosophies (Schouten & McAlexander, 1995; Kozinets, 2001; Fuschillo, 2020). In particular, Muñiz and O’Guinn (2001) conceptualized brand communities as non-geographic communities characterized by a consciousness of kind, rituals and traditions, and a sense of moral responsibility, while McAlexander et al. (2002) and Schau et al. (2009) argued that members of brand communities share identity, meaning, knowledge, and emotional commitment through collective practices and collectively create brand value. Therefore, brand fandom can be defined not as a mere collection of individuals who prefer a brand, but as a group of active consumers who define themselves through the brand, bond with others who share similar passions, and jointly produce brand meaning and value through acts of sharing, advocacy, and creation (Laroche et al., 2012; Y. Xu & Hong, 2022; Cheng et al., 2023; Preiksaitis & Pearson, 2025).

Brand fandom cannot be fully explained by satisfaction or traditional concepts of loyalty alone (Cheng et al., 2023). While loyalty focuses on sustained relationships at the individual level—such as repeat purchases, attitudinal preferences, and emotional attachment (Fullerton, 2005; Damaschi et al., 2025; Na et al., 2023)—brand fandom is a relational and collective phenomenon that involves social bonds with other consumers, collective rituals and practices, and the co-creation of brand meaning and value (Muñiz & O’Guinn, 2001; Fuschillo, 2020). Furthermore, while brand love is a concept that describes passionate emotional attachment to a specific brand and the integration of self-identity at the individual level (Shimp & Madden, 1988; Carroll & Ahuvia, 2006; Batra et al., 2012), brand fandom locates that relationship in a collective (Escalas & Bettman, 2005; Jin & Park, 2025).

This relational and collective nature does not remain merely an abstract attitude but manifests as concrete and voluntary pro-brand behavior. Research on brand communities documents both the social diffusion of brand-related content to others and the active production of brand meaning (Schau et al., 2009; Muñiz & Schau, 2005). Accordingly, this study categorizes brand fandom behaviors into sharing and advocacy behaviors and creation behaviors. While both types of behavior share the common characteristics of being pro-brand, voluntary, and active, they differ in that sharing and advocacy behaviors focus on social diffusion within networks, whereas creation behaviors focus on the productive transformation of brand meanings and usage patterns.

Sharing and advocacy behaviors are defined as pro-brand behaviors in which consumers voluntarily disseminate brand-related content, experiences, and information to others, recommend the brand, or defend it in critical situations (J. Kim et al., 2021; Stokburger-Sauer et al., 2012; Xie et al., 2017). This includes information dissemination—such as social media posts, sharing personal experiences, and voluntary information sharing—as well as active recommendations, solicited opinions, and defending against or correcting negative evaluations (Choi & Burnham, 2021; Babić Rosario et al., 2016; de Vries et al., 2012). Sharing and advocacy behaviors emerge as consumers transform the emotional and cognitive engagement they have formed with a brand into social practices (Hollebeek et al., 2014), and they contribute to the brand’s social visibility and reputation by disseminating brand value within individuals’ social networks (Lim & Brown-Devlin, 2021; Xie et al., 2017). Within brand communities, these behaviors appear as evangelizing and justifying practices (Schau et al., 2009).

Creation behaviors are defined as an active, pro-brand behavior in which consumers voluntarily produce and modify content, meanings, and usage patterns related to a brand (Muñiz & O’Guinn, 2001; Schau et al., 2009). It takes the form of content-based creation—the production and sharing of user-generated content such as reviews, analyses, photos, and videos (Malodia et al., 2024)—and meaning-based creation, which assigns new symbolic meanings and contexts of use beyond a product’s original purpose (Muñiz & O’Guinn, 2001; Muñiz & Schau, 2005), and use-based creation, which involves developing know-how, customizations, and usage routines for products and services (Schau et al., 2009). Creation behaviors serve as a behavioral expression of brand engagement (Hollebeek & Macky, 2019) and are a core aspect of fandom, in which consumers do not merely accept the perceptions and meanings formed at brand touchpoints but reinterpret and expand upon them in their own way (Xie et al., 2017). Creative practices of this kind—customizing, grooming, commoditizing, and documenting—have been documented across brand communities (Schau et al., 2009).

#### 2.2.1. Self-Transcendent Emotion and Brand Fandom Behaviors

The experience of self-transcendent emotion does not remain confined to the realm of personal experience but can extend to an outward motivation to share or express that experience with others (Zickfeld et al., 2019). Rimé (2009), in a theoretical and empirical review of the social sharing of emotion, documents that emotional experiences are habitually recounted to others, which is how self-transcendent emotion promotes sharing and advocacy behaviors (Stellar et al., 2017). Online content evoking high-arousal positive emotions such as awe is shared more actively (Berger & Milkman, 2012), emotional experiences described as being moved strengthen positive word of mouth, loyalty, and commitment (Griessmair et al., 2022), and brand awe directly promotes the desire to save and share brand experiences (J. Kim et al., 2021), with emotional engagement leading to positive word of mouth and advocacy intentions (Fullerton, 2005; Wang et al., 2021; Rahi et al., 2026).

The experience of self-transcendence extends beyond sharing and advocacy to creation behaviors. Emotional experiences foster behavioral motivation toward connecting with others (Stellar et al., 2017; Zickfeld et al., 2019), and in digital environments this motivation is transformed into expressions such as reviews, images, videos, stories, and usage tips, through which consumers jointly create brand value by storytelling, customization, and sharing usage methods (Habibi et al., 2014; Prahalad & Ramaswamy, 2004). Drawing on the above discussion, the following hypotheses were proposed.

**H7.**  *Self-transcendent emotion has a positive (+) effect on sharing and advocacy behaviors.*

**H8.**  *Self-transcendent emotion has a positive (+) effect on creation behaviors.*

#### 2.2.2. Self-Brand Connection and Brand Fandom Behaviors

The stronger the self-brand connection, the more a person’s response to a brand extends beyond personal preferences or attitudes to a behavioral tendency to support and share the brand—which is linked to their identity—with others (Choi & Burnham, 2021; Stokburger-Sauer et al., 2012). Belk (1988), in developing the concept of the extended self, argues that consumers treat possessions as part of who they are; brand-related information and experiences accordingly take on self-expressive meaning, which is how self-brand connection promotes sharing and advocacy behaviors (Escalas & Bettman, 2005). Identification strengthens brand loyalty and brand advocacy behavior, in online brand communities as elsewhere (Stokburger-Sauer et al., 2012; Mousavi et al., 2017); self-expressive brands increase positive word of mouth and brand loyalty (Carroll & Ahuvia, 2006), and consumers’ cognitive, emotional, and behavioral activities related to brands in social media are associated with self-brand connection and brand usage intentions (Hollebeek et al., 2014). Brand identification in social media-based brand communities promotes community participation, which in turn leads to brand love and, through it, to brand-related information-sharing behaviors (C. T. Lee & Hsieh, 2022).

The self-brand connection also serves as an important basis for explaining creation behaviors. Consumers construct and express their self-concept through brands, and the more closely a brand is linked to the self, the more it becomes a symbolic resource for self-expression and self-extension rather than merely an object of consumption (Fournier, 1998; Escalas & Bettman, 2005). When this connection is strengthened, consumers do not merely accept brand meanings but tend to interpret and expand upon them using their own language and methods (Muñiz & Schau, 2005; Dessart et al., 2015), acting as producers of brand-related content rather than observers of it (Muntinga et al., 2011). The more consumers incorporate brand pages into their self-concept, the more actively they create brand-related content (Nikolinakou et al., 2021), and members of brand communities collectively produce brand meaning and value through storytelling, customization, documentation, and the reconfiguration of usage patterns (Schau et al., 2009; Muñiz & O’Guinn, 2001). Based on this, the following hypotheses were proposed.

**H9.**  *Self-brand connection has a positive (+) effect on sharing and advocacy behaviors.*

**H10.**  *Self-brand connection has a positive (+) effect on creation behaviors.*

### 2.3. Need for Self-Expression

The need for self-expression refers to a motivational tendency for consumers to outwardly express elements of their self-identity—such as personality traits, preferences, values, beliefs, opinions, or attitudes—that are difficult to observe directly (H. S. Kim & Sherman, 2007; Morgan & Townsend, 2022). Self-expression makes internal attributes more concrete and self-defining, thereby reinforcing one’s sense of identity (H. S. Kim & Sherman, 2007), and self-expressive consumption yields not only utilitarian benefits such as identity formation and maintenance but also hedonic benefits derived from the act of expression itself (Morgan & Townsend, 2022). In a consumption context, the need for self-expression serves as a key motivational foundation for consumers’ brand selection and usage. Consumers use the symbolic meanings of brands to construct and express their self-identity and to differentiate themselves from others (Escalas, 2004; Escalas & Bettman, 2005). Consumers with a stronger need for self-expression accordingly participate more actively in self-expressive word-of-mouth arising from consumption experiences (Saenger et al., 2013).

Therefore, at equal levels of self-transcendent emotion and self-brand connection, consumers differ in how far they externalize those responses: for those high in the need for self-expression, brand-related sharing, recommendation, advocacy, and content creation are not merely information dissemination or participation but visible acts that reveal identity, tastes, and values (H. S. Kim & Sherman, 2007; Morgan & Townsend, 2022; Escalas & Bettman, 2005; Chernev et al., 2011). Previous studies have shown that identity and self-expansion motives promote sharing and creative activity in social media environments (Muntinga et al., 2011; Nikolinakou et al., 2021). They have also shown that paths of this kind are themselves conditional: the effect of brand loyalty on word of mouth depends on self-brand connection (Eelen et al., 2017), and self-expression moderates the effect of oppositional loyalty on brand experiences and self-brand connection in online brand communities (Chen & Liao, 2023). Based on this prior research, the following hypothesis was proposed.

**H11.**  *The level of need for self-expression will moderate the effects of self-transcendent emotion and self-brand connection on brand fandom behaviors.*

## 3. Research Methods

### 3.1. Research Model

**Figure 1:**
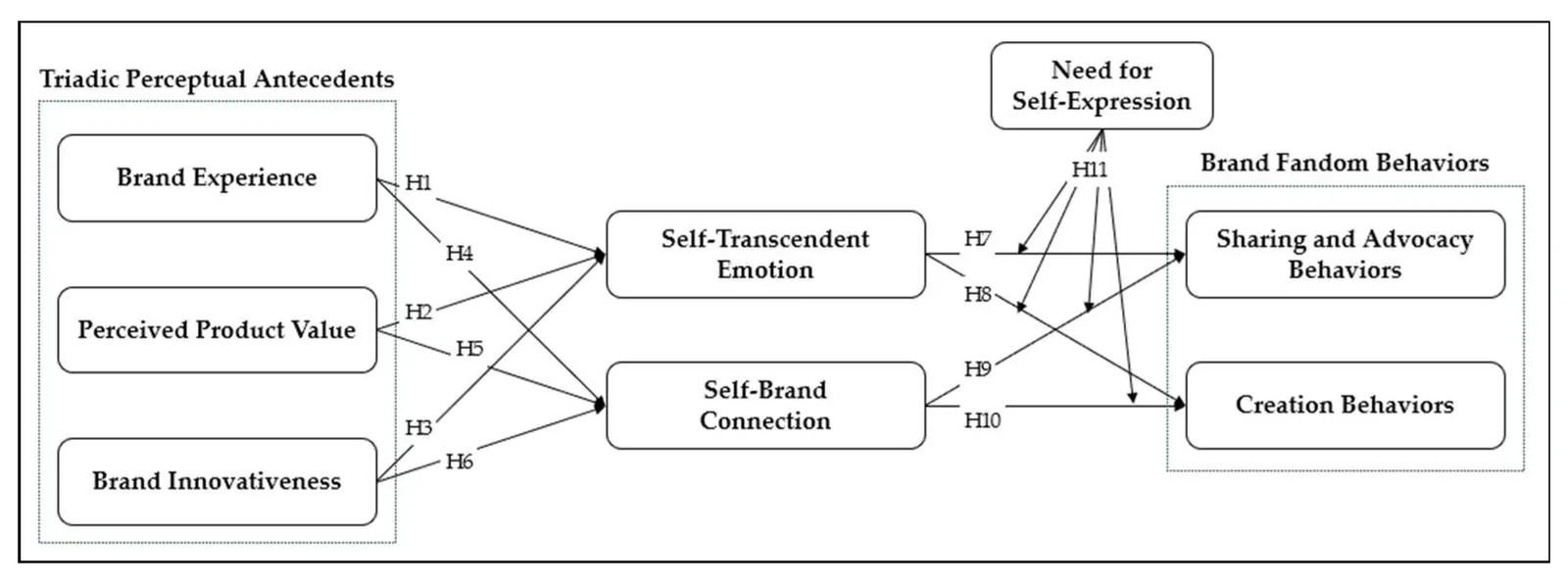
Research Model.

Based on the preceding literature review, this study examines how three brand-related perceptual antecedents—brand experience, perceived product value, and brand innovativeness—influence sharing and advocacy behaviors and creation behaviors through self-transcendent emotion and self-brand connection. It also examines whether the need for self-expression moderates these second-stage relationships. Figure 1 presents the proposed relationships, which were estimated simultaneously within a single structural equation model. Each path in Figure 1 corresponds to a hypothesis developed in Section 2, where the reasoning underlying it is stated. Because the two psychological pathways are what the study sets out to test, the hypothesized model specifies the antecedents as reaching the behaviors through an affective response worth communicating or an identity stake in the brand’s standing. Self-transcendent emotion and self-brand connection were specified as parallel rather than sequential mediators because they represent conceptually distinct affective and identity-based responses, and the existing literature provides no clear basis for assuming a causal ordering between them. The moderator is specified on the second stage alone, since the need for self-expression bears on whether an internal response is externalized rather than on whether it forms. Each relationship is read as monotonic within the range observed, the accounts on which the hypotheses draw specifying direction rather than functional form.

This study selected Tesla, a high-technology brand in the electric vehicle market, as its empirical context. Tesla’s vehicle features stimulate a wide range of sensory, behavioral, and intellectual brand experiences, while perceptions of its quality and of its social image correspond to the two product-value dimensions measured here. Furthermore, Tesla is widely recognized as an innovative pioneer in this market. As a brand where sharing, advocacy, and creation behaviors are routinely observed, Tesla provides a context in which the two dependent variables of this study can be meaningfully measured (Barr, 2025; L. Lee, 2025; Tesla Owners UK, 2026). The constructs in this model are not equally activated in every brand context: self-transcendent emotion presupposes a brand that can be perceived as exceeding what the consumer expects the category to be capable of, and creation behaviors presuppose a product whose use can be reconfigured by the consumer. Tesla was selected because both conditions hold, giving a setting in which the process of interest should be clearly observable (Eisenhardt, 1989).

### 3.2. Measurement Variable and Data Collection

To validate the proposed research model, this study designed and conducted a structured survey. The measurement items are summarized in Table 1; each construct was measured from the definition given in Section 2. Brand experience was measured with the scale of Brakus et al. (2009), on its sensory, behavioral, and intellectual dimensions. Perceived product value was measured with the PERVAL scale of Sweeney and Soutar (2001), on its quality and social value dimensions. Brand innovativeness was measured with the scale of Shams et al. (2015), as the extent to which consumers perceive a brand as a creative and future-oriented symbolic actor.

Two of these constructs were measured with a subset of the dimensions in the source scale. For brand experience, the affective dimension of the Brakus et al. (2009) scale was not administered. Brakus et al. (2009, p. 54) distinguish brand experience from emotional relationship constructs and note that brand experiences “may result in emotional bonds,” emotion being an outcome of experience rather than a component of it. The affective response is therefore placed downstream, as self-transcendent emotion, and brand experience is measured on its sensory, behavioral, and intellectual facets. For perceived product value, quality/performance and social value were administered. Emotional value was excluded for the same reason; price/value-for-money was excluded because it is a judgment about a transaction rather than about the brand, and the sample comprises both owners and prospective buyers, for whom it would not share a common referent. These exclusions narrow the construct as measured here rather than treat the omitted dimensions as redundant.

Self-transcendent emotion was measured following Zickfeld et al. (2019) and J. Kim et al. (2021), as the emotional elevation response elicited by brand stimuli. Self-brand connection was measured with the scale of Escalas and Bettman (2005). The need for self-expression was measured with the self-expressive brand scale of Carroll and Ahuvia (2006). A further departure concerns the moderator. The self-expressive brand scale of Carroll and Ahuvia (2006) measures whether a particular brand expresses the consumer’s self, whereas this study requires an individual-difference moderator; the referent was therefore shifted from the brand to the respondent’s general consumption disposition, consistent with the conceptualization of self-expression as a motivational tendency in H. S. Kim and Sherman (2007) and Morgan and Townsend (2022), and the questionnaire section instructed respondents to answer on the basis of their usual disposition rather than any specific brand or product.

Items for sharing and advocacy behaviors and for creation behaviors were assembled from prior research; the source of each item and the adaptation applied to it are given in Appendix A. Most items were adapted from published measurement items; four were developed for this study from a construct or a community practice that the source describes but does not operationalize as a survey item. Appendix A marks which of the two applies to each item.

The initial survey consisted of a total of 37 items. Of these, 36 were retained after the confirmatory factor analysis reported in Section 4.1. Two items per dimension were administered rather than the full item set of each source scale, in order to keep the questionnaire to a length suited to online panel administration; for brand experience, the item omitted from each dimension was the negatively worded one. Its psychometric properties were re-established in this sample rather than assumed from the source scales (Section 4.1). The specific items for each construct are listed in Table 1. Data analysis was conducted using SPSS 28.0 for descriptive statistics, while structural equation modeling based on AMOS 25.0 was used to test the structural path hypotheses. Regression analysis in SPSS 28.0 and PROCESS macro v4.2 (Hayes, 2022) were used to test the moderating effect of the need for self-expression. The measurement invariance, multi-group, and robustness analyses reported in Section 4.1, Section 4.2, Section 4.3 and Section 4.4 were conducted in R using the lavaan package, version 0.6-17 (Rosseel, 2012). The source of each scale, the adaptation applied to it, and the translation procedure are documented in Appendix A.

### 3.3. Demographic Information of the Data

The data were collected in February 2026 through online panel companies in Republic of Korea and the United States. The questionnaire was administered in Korean or in English according to the respondent’s country of residence; the translation procedure and the evidence for equivalence between the two versions are given in Appendix A. All items were measured using a 5-point Likert scale (1 = Strongly disagree, 5 = Strongly agree). All participants provided informed consent. The target respondents for this study were Tesla owners who drive their vehicles at least once a week, and prospective buyers who do not currently own a Tesla but have a sustained interest in the Tesla brand and are considering a future purchase. Respondents who expressed no interest in Tesla were excluded from the survey after the first screening question.

During the data collection process, we used technical filtering to block bot responses and removed invalid responses based on criteria such as abnormal response times and repetitive responses following the same pattern. As a result, the final analysis sample consisted of 635 respondents, comprising 313 from Republic of Korea and 322 from the United States. The demographic characteristics of the respondents are presented in Table 2.

## 4. Results

### 4.1. Measurement Model Assessment and Invariance

Prior to hypothesis testing, confirmatory factor analysis was conducted to evaluate the reliability, convergent validity, and discriminant validity of the measurement model. In the initial confirmatory factor analysis, one item in the sensory dimension of brand experience exhibited a low squared multiple correlation (SMC = 0.377), and since the average variance extracted (AVE) for that construct fell short of the 0.50 criterion, this item was excluded from the final analysis to improve convergent validity. The final measurement model estimated after item exclusion exhibited good model fit (χ^2^/df = 1.569, GFI = 0.932, AGFI = 0.919, NFI = 0.956, TLI = 0.982, CFI = 0.984, RMSEA = 0.030). In the final measurement model, all standardized factor loadings ranged from 0.649 to 0.939, exceeding the recommended threshold of 0.60, and all loadings were statistically significant at the *p* < 0.001 level. Composite reliability (CR) ranged from 0.849 to 0.946, and average variance extracted (AVE) ranged from 0.530 to 0.801, meeting the criteria of 0.70 and 0.50, respectively, thereby establishing convergent validity (Hair et al., 2019). Furthermore, Cronbach’s α values for all constructs ranged from 0.848 to 0.947, all exceeding 0.70, confirming internal consistency. The results for reliability and convergent validity are presented in Table 3.

Because brand experience is conceived as a multidimensional construct, removing an indicator raises a question of content validity that a statistical criterion alone cannot settle. The excluded and the retained sensory item address the same facet—the first asks whether the brand makes a strong impression on the senses, the second whether the brand is sensorially interesting—so the sensory facet is not left unmeasured, although it is now carried by a single indicator. Excluding the item raised the average variance extracted for brand experience from below the 0.50 threshold to 0.530, while the construct continues to span the sensory, behavioral, and intellectual facets specified in Section 3.2. The narrower representation of the sensory facet is acknowledged as a limitation of the measurement.

The need for self-expression, which enters the model as a moderator rather than as part of the structural measurement model, was additionally examined as an eighth factor. It met the same criteria (CR = 0.906, AVE = 0.764) and correlated 0.169 with self-brand connection, indicating that the moderator is empirically distinct from the identity-based mediator whose effect it conditions.

Furthermore, an analysis of the AVE values and correlation coefficients among the latent variables (see Table 4) confirmed that the square root of the AVE for each construct was greater than its correlations with all other constructs, indicating that discriminant validity was established (Fornell & Larcker, 1981).

Because the sample combines respondents from two countries and two ownership groups, measurement invariance was tested before the structural model was estimated (Vandenberg & Lance, 2000; Cheung & Rensvold, 2002). Configural, metric, and scalar models were estimated separately for the Korean and U.S. subsamples and for owners and non-owners. As reported in Table 5, imposing equality of factor loadings and then of item intercepts did not significantly degrade fit in either grouping, and the change in comparative fit index did not exceed 0.001 at any step, well within the 0.010 criterion recommended by Cheung and Rensvold (2002). Full scalar invariance therefore holds across both country–language groups and ownership status, indicating that the constructs carry the same meaning and the same metric in each group and that structural parameters can be meaningfully compared and pooled across them.

Common method bias was assessed with two of the procedures described by Podsakoff et al. (2003). In Harman’s single-factor test, the first unrotated component accounted for 33.17% of the total variance, below the conventional 50% threshold, and seven components returned eigenvalues above one, corresponding to the seven constructs of the measurement model. A common latent factor was then added to the confirmatory factor model; standardized loadings shifted by 0.034 on average, well below the 0.20 difference conventionally read as evidence of method bias. Neither diagnostic indicated substantial common method bias.

### 4.2. Results of Hypothesis Testing

Structural equation modeling was conducted to test the research hypotheses. The structural model exhibited good fit (χ^2^/df = 1.690, GFI = 0.925, AGFI = 0.913, NFI = 0.952, TLI = 0.978, CFI = 0.980, RMSEA = 0.033), and all ten paths from H1 to H10 were supported; the estimates are reported in Table 6. Two features of the coefficient pattern bear on interpretation. First, the antecedents are ordered differently for the two mediators. Brand innovativeness is the strongest input to self-transcendent emotion (β = 0.244), roughly twice the effect of brand experience and close to three times that of perceived product value, whereas brand experience is the strongest input to self-brand connection (β = 0.302). These orderings should be read descriptively: within-equation Wald tests on the unstandardized coefficients distinguish brand innovativeness from perceived product value for self-transcendent emotion (χ^2^(1) = 7.00, *p* = 0.008), but brand innovativeness and brand experience are not statistically distinguishable in either equation (*p* = 0.168 and *p* = 0.326), and the apparent crossover between the two equations is not significant (χ^2^(1) = 2.98, *p* = 0.084). The relative pattern is therefore reported as an observation requiring further test rather than as an established difference. Second, the internal responses are far less well explained than the behaviors they produce: the three antecedents account for 11.3% of the variance in self-transcendent emotion and 30.1% in self-brand connection, while the two mediators together account for 60.0% of the variance in sharing and advocacy behaviors and 46.2% in creation behaviors. The model therefore accounts for substantially more variance in the behaviors than in the internal responses that precede them.

### 4.3. Results of Mediation Analysis

To verify the mediating effects of self-transcendent emotion and self-brand connection, we applied the bootstrap procedure. We assessed the significance of each indirect effect based on the 95% confidence interval calculated through 5000 resampling runs, and an indirect effect was considered significant when the confidence interval did not include 0. Since this study focused on explaining the emotional and identity-based mediating pathways through which brand-related perceptions are transformed into fandom behaviors via self-transcendent emotion and self-brand connection, the basic research model established a mediating structure that did not include direct paths between the three antecedent variables and fandom behaviors. As shown in Table 7, the confidence intervals for all 12 indirect paths did not include 0, indicating that they were statistically significant.

The two mediators show different relative orderings among the indirect effects they transmit, subject to the qualification noted in Section 4.2. Among the six paths running through self-transcendent emotion, brand innovativeness carries the largest indirect effect (0.136 to sharing and advocacy behaviors; 0.126 to creation behaviors), roughly three times the effect of perceived product value. Among the six paths running through self-brand connection, brand experience is the strongest (0.173 and 0.152, respectively), which mirrors the pattern in the direct effects reported in Section 4.2. The full estimates are given in Table 7.

Because the hypothesized model constrains the three antecedents to operate entirely through the two mediators, we additionally estimated a partially mediated model in which direct paths from brand experience, perceived product value, and brand innovativeness to both behavioral outcomes were freely estimated. This less constrained model fits better than the hypothesized model (Δχ^2^(6) = 33.641, *p* < 0.001; CFI 0.984 versus 0.982; SRMR 0.028 versus 0.037; both estimated in lavaan rather than AMOS). All twelve indirect effects remained significant under this less constrained specification (e.g., brand experience → self-brand connection → sharing and advocacy behaviors = 0.142, 95% CI [0.097, 0.197]; brand innovativeness → self-transcendent emotion → sharing and advocacy behaviors = 0.123, 95% CI [0.076, 0.174]), indicating that the mediating pathways are robust to model specification. Following Zhao et al. (2010) and Rucker et al. (2011), we interpret the four significant direct paths as complementary mediation and the two non-significant ones as indirect-only mediation, rather than as evidence against mediation: the significance of a direct path does not qualify an indirect effect, and the full-versus-partial mediation dichotomy has been argued to be sample-size dependent and of limited value as a criterion. The theoretically specified indirect model is therefore retained for hypothesis testing, and the alternative specification is reported in Appendix B for transparency. Sequential specifications in which one mediator predicts the other were also estimated in both directions; neither added path was significant (β = −0.030 and −0.038, both *p* = 0.441) and neither improved fit (Δχ^2^(1) = 0.591), which provides no evidence favoring either sequential ordering.

### 4.4. Moderation and Robustness Analyses

**Figure 2:**
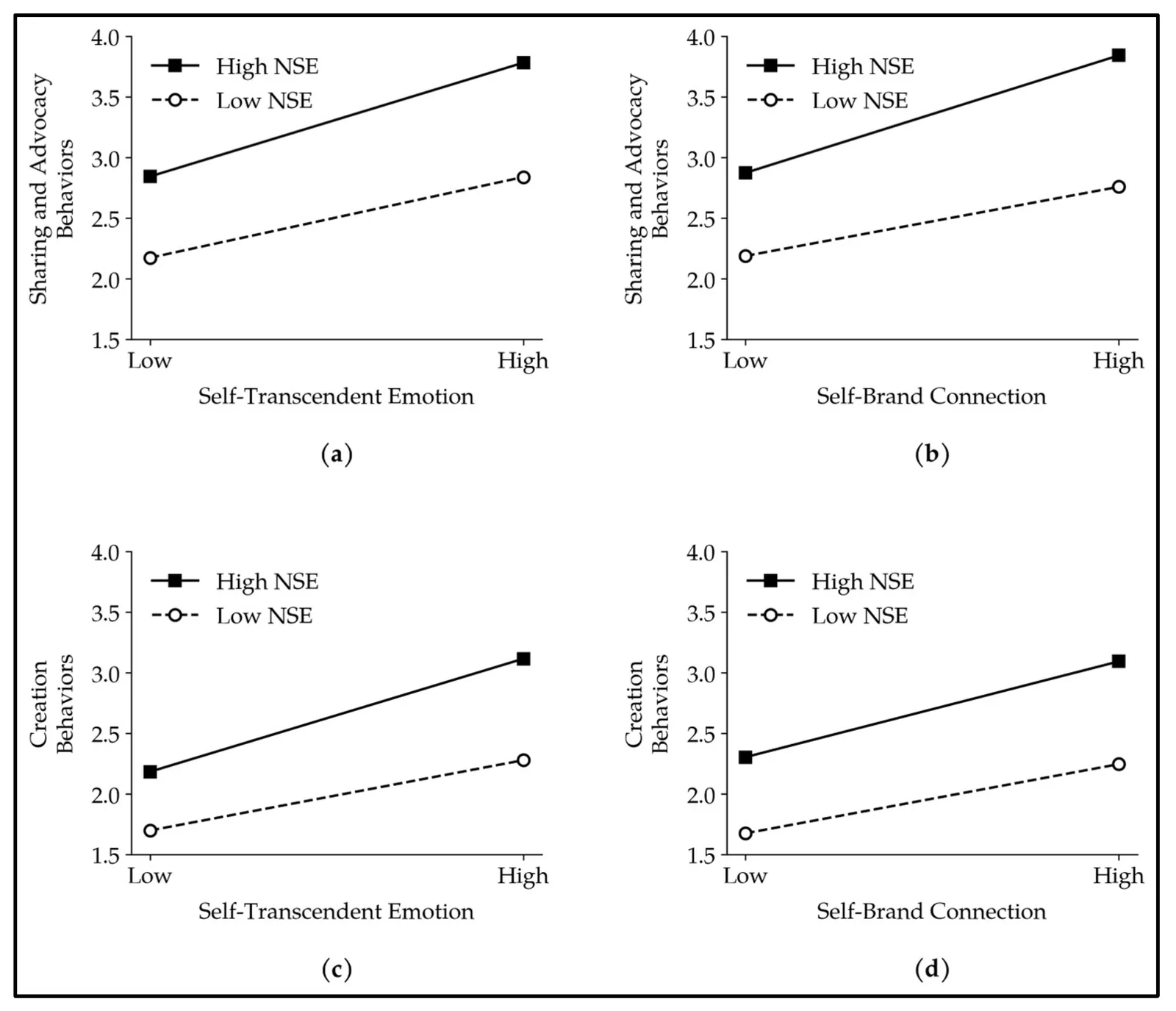
Simple slope analyses for the moderating role of need for self-expression (NSE) on four second-stage paths. (**a**) Self-transcendent emotion to sharing and advocacy behaviors; (**b**) self-brand connection to sharing and advocacy behaviors; (**c**) self-transcendent emotion to creation behaviors; (**d**) self-brand connection to creation behaviors.

The moderating effect of the need for self-expression was tested with PROCESS Model 1. The moderating variable, the need for self-expression, was measured using three items and demonstrated good internal consistency (Cronbach’s α = 0.904). This study tested the moderating effect of the need for self-expression on each of the four second-stage pathways through which self-transcendent emotion and self-brand connection influence sharing and advocacy behaviors and creation behaviors. As shown in Table 8, the interaction term was statistically significant in all four pathways, supporting H11. Specifically, the interaction term between self-transcendent emotion and the need for self-expression was significant for sharing and advocacy behaviors (B = 0.103, *p* = 0.010, 95% CI [0.025, 0.181]) and creation behaviors (B = 0.133, *p* = 0.002, 95% CI [0.048, 0.217]). Furthermore, the interaction term between self-brand connection and the need for self-expression also had a significant positive effect on both sharing and advocacy behaviors (B = 0.166, *p* < 0.001, 95% CI [0.090, 0.242]) and creation behaviors (B = 0.091, *p* = 0.035, 95% CI [0.007, 0.176]). According to the simple slope analysis in Figure 2, the slope for the group with a high need for self-expression was steeper (positive) than that for the group with a low need for self-expression across all four paths. This implies that the higher the need for self-expression, the stronger the influence of these two mediating variables on fandom behaviors.

Because the four moderation tests reported in Table 8 examine each mediator separately, the moderation was additionally re-estimated in a single model containing both mediators, their interactions with the need for self-expression, and the three antecedents, so that the index of moderated mediation could be computed (Hayes, 2015, 2022). All four second-stage interaction terms remained significant in this joint specification (self-transcendent emotion × need for self-expression: B = 0.084, *p* = 0.012 for sharing and advocacy behaviors and B = 0.125, *p* = 0.001 for creation behaviors; self-brand connection × need for self-expression: B = 0.180, *p* < 0.001 and B = 0.097, *p* = 0.012), and the bootstrap confidence intervals for all twelve indices of moderated mediation excluded zero, indicating that every conditional indirect effect strengthens as the need for self-expression increases. Full results are reported in Appendix C.

Three further analyses were conducted to assess the robustness of the results. First, to examine whether pooled estimation obscures heterogeneity between the Korean and U.S. subsamples or between owners and non-owners, multi-group structural models were estimated on the scalar-invariant measurement model established in Section 4.1. Constraining all ten structural paths to equality across groups did not significantly worsen model fit for either grouping (country: Δχ^2^(10) = 4.276, *p* = 0.934; ownership: Δχ^2^(10) = 7.535, *p* = 0.674). This provides no evidence that these relationships differ across the country–language groups or across ownership groups, which supports estimating the model on the combined sample. The multi-group test above constrains the ten structural paths, which do not include the moderation specified in H11, so the moderation was compared across countries separately. The need for self-expression met full scalar invariance across the Korean and U.S. subsamples (metric: Δχ^2^(2) = 0.324, *p* = 0.851; scalar: Δχ^2^(2) = 1.500, *p* = 0.472; ΔCFI = 0.000), and its latent mean did not differ between them (difference = 0.059, *p* = 0.375). Allowing all second-stage interaction terms to vary by country did not improve fit over a model in which they are common to both (sharing and advocacy behaviors: F(5, 623) = 0.533, *p* = 0.752; creation behaviors: F(5, 623) = 0.384, *p* = 0.860), and no three-way term reached significance. The conditional slopes are correspondingly close in the two subsamples (for example, self-brand connection → sharing and advocacy behaviors at higher self-expression: 0.158 in Korea and 0.203 in the United States).

Second, because the sample is predominantly male (95.3%), two checks were performed. Gender was entered as a covariate on all four endogenous constructs; its effect was non-significant in every case (|β| ≤ 0.036, *p* ≥ 0.227) and the ten structural coefficients were unchanged to the third decimal. The model was then re-estimated on the male subsample alone (n = 605); fit remained good (χ^2^/df = 1.618, CFI = 0.981, TLI = 0.979, RMSEA = 0.032) and all ten paths remained significant with coefficients closely tracking the same model fitted to the full sample in lavaan (brand experience → self-brand connection: 0.305 versus 0.301; self-transcendent emotion → sharing and advocacy behaviors: 0.508 versus 0.520). The principal estimates were therefore robust to excluding the small female subsample, although the sample constrains generalization to female consumers.

Third, because all hypotheses specify positive linear relationships, quadratic terms were added to each structural equation, in a supplementary regression on construct scores, to test for curvilinear effects. Their addition did not significantly improve explained variance in any of the four equations (self-transcendent emotion: F(3, 628) = 0.576, *p* = 0.631; self-brand connection: F(3, 628) = 0.226, *p* = 0.878; sharing and advocacy behaviors: F(2, 630) = 1.895, *p* = 0.151; creation behaviors: F(2, 630) = 1.668, *p* = 0.189), and no individual quadratic coefficient reached significance. The linear specification underlying the hypotheses is therefore not contradicted by the data within the range observed.

## 5. Discussion

This study empirically analyzed the pathways through which brand experience, consumers’ perceptions of product value, and perceptions of brand innovativeness lead to sharing and advocacy behaviors and creation behaviors via self-transcendent emotion and self-brand connection in the context of high-tech brands. It also examined how the downstream pathway—where these two mediating variables translate into fandom behaviors—differs depending on the level of the need for self-expression.

First, all three brand perception factors were found to have a significant positive effect on self-transcendent emotion (STE) and self-brand connection (SBC). This implies that experiences with the brand, perceived value derived from product use, and brand innovativeness can all function as psychological input factors in the formation of fandom. These results align with the discussion by Brakus et al. (2009), who argued that brand experiences can elicit sensory, affective, behavioral, and intellectual responses in consumers and serve as the experiential foundation for the formation of consumer–brand relationships. Furthermore, this study extends the PERVAL research by Sweeney and Soutar (2001)—which proposed that perceived product value, as a multidimensional construct encompassing quality/performance, emotional, social, and price/value-for-money dimensions, is instrumental in explaining consumer attitudes and behavior—to the context of brand fandom. Furthermore, the finding that perceived brand innovativeness significantly influences both STE and SBC aligns with the discussion by Henard and Dacin (2010) that innovation reputation can positively influence consumer excitement, loyalty, and corporate image, as well as the finding by Shams et al. (2017) that brands perceived as more innovative attract significantly higher purchase intention.

Examining the relative patterns of the antecedent variables, perceived brand innovativeness showed the largest point estimate for self-transcendent emotion (β = 0.244), while brand experience showed the largest for self-brand connection (β = 0.302). This suggests that, in the context of high-tech brands, perceived brand innovativeness is relatively closely linked to self-transcendent emotional responses such as awe, inspiration, and wonder, whereas direct and repeated experiences with the brand may play a stronger role in the process by which consumers connect the brand to their self-identity. One reading, consistent with the two processes proposed in Section 2, is that emotional elevation depends on the brand being appraised as exceeding what the consumer expected the category to be capable of, whereas identity integration depends on accumulated, personally held material that the consumer can use to define the self. Because brand innovativeness and brand experience are not statistically distinguishable within either equation (Section 4.2), this interpretation is offered as a hypothesis for designs able to test the two processes directly rather than as a finding of the present study. The low proportion of variance explained in self-transcendent emotion (*R*^2^ = 0.113) may be read in the same light: a response of this kind appears to depend considerably on the individual consumer and on the particular encounter, so that brand-level perceptions establish conditions under which it may arise rather than accounting for it directly. Self-brand connection, which describes a more settled relation between brand and self-concept, is correspondingly better explained (*R*^2^ = 0.301).

Second, all 12 indirect effects—in which three brand perception factors lead to sharing and advocacy behaviors and creation behaviors via self-transcendent emotion and self-brand connection—were found to be statistically significant. This implies that psychological transformation pathways, namely self-transcendent emotional experiences and self-brand connection, play a crucial role in the process by which consumers’ brand perceptions manifest as fandom behaviors. The mediation results via self-transcendent emotion align with the discussion by Schouten et al. (2007), who noted that intense brand experiences can lead to transcendent customer experiences and active brand advocacy, as well as the findings of Stellar et al. (2017) and Zickfeld et al. (2019), who argued that self-transcendent emotions activate communal bonds and motivations for prosocial participation. The mediation results via self-brand connection also align with the findings of Stokburger-Sauer et al. (2012), Nikolinakou et al. (2021), and C. T. Lee and Hsieh (2022), which show that consumers with strong brand identification and self-brand connection participate more actively in brand advocacy, recommendations, and content creation. In particular, since behaviors such as evangelizing, documenting, and customizing—which Schau et al. (2009) identified as value-creating practices within brand communities—are linked to the conceptual foundations of sharing, advocacy, and creation behaviors in this study, the results demonstrate that the two psychological pathways—emotional elevation and identity integration—function as a parallel mediating structure explaining fandom behaviors.

These indirect effects do not, however, establish that the three perceptions operate only through the two mediators: the partially mediated model reported in Section 4.3 fits better, and four of its six direct paths are significant, so the pattern is one of complementary rather than complete mediation. Which perception requires transformation also differs. Brand experience shows no direct association with sharing and advocacy behaviors once the two mediators are included, whereas perceived product value and brand innovativeness retain direct associations with that outcome, which is consistent with value and innovativeness perceptions being conveyable as informational content in their own right while experiential perception requires affective or identity-based transformation before it is externalized.

A further observation concerns the two mediators themselves. Both predict the two behavioral outcomes strongly, yet they correlate only weakly with each other (r = 0.151), which suggests that they operate with a considerable degree of independence in this context. This is consistent with the distinction drawn in the Introduction, that the two describe different directions of the consumer–brand relation rather than two intensities of the same relation. A correlation of this size does not establish that a consumer follows only one route, but it does indicate that the two responses vary largely independently in this sample, which a single-mediator specification would not have made visible. One consequence for the literature is that a finding obtained with either mediator alone may not extend to the other route, even though both predict the same behaviors.

Third, the need for self-expression was found to strengthen all four downstream pathways through which the two mediating variables translate into fandom behaviors. This implies that even when experiencing the same level of emotional and identity-based reactions, consumers with a high need for self-expression are more likely to express these reactions through external behaviors—such as sharing, recommending, advocating, and content creation—rather than keeping them as internal reactions. These results are consistent with H. S. Kim and Sherman’s (2007) argument that individuals with a high need for self-expression tend to visualize their values and preferences through external actions, and they align with Morgan and Townsend’s (2022) assertion that self-expressive consumption involves not only the utilitarian need for identity construction but also the hedonic satisfaction derived from the act of expression itself. Furthermore, these findings align with the empirical evidence presented by Saenger et al. (2013), the finding by Carroll and Ahuvia (2006) that consumers of self-expressive brands exhibit stronger positive word-of-mouth behavior, and the results by Nikolinakou et al. (2021) showing that consumers with strong self-expression and self-expansion motives participate more actively in sharing and content creation on social media brand pages. These studies concern related but distinct forms of self-expression; Carroll and Ahuvia’s (2006) result in particular is about brands perceived as self-expressive rather than about consumers disposed to self-expression. The present study treats consumption-related self-expression as a general individual disposition and locates its role specifically in the conversion of emotional and identity-based responses into behavior. Consequently, this study demonstrates that the need for self-expression is not merely a variable of individual difference but can act as a motivational reinforcement that translates emotional and identity-based responses into external behavior.

Two features of this result bear on the structure of the model. The moderator strengthened all four second-stage paths, so the two routes, distinct as they are, share the same expressive condition on the way to action; and it is empirically distinct from the identity-based mediator whose effect it conditions (Section 4.1). The formation of an internal fan response and its conversion into visible behavior are therefore better treated as two stages than as one.

This bears on the cultural literature from which the moderator is drawn. H. S. Kim and Sherman (2007) report that European Americans value self-expression more highly than East Asian Americans, and that expressing a choice affected commitment to it for the former but not the latter, which would predict a weaker expressive route in the Korean subsample. No such difference was observed here, either in the level of the need for self-expression or in its moderating effect (Section 4.4). The samples are not comparable to those of that study, however: respondents in both countries were selected for sustained interest in a single global technology brand, so the finding indicates that the expressive condition operated similarly within a brand-interested population rather than that the cultural difference reported at the population level does not hold.

## 6. Conclusions

### 6.1. Implications

Brand fandom has drawn increasing attention as a form of consumer–brand relationship that goes beyond repeat purchase and favorable attitude. This study shows that it operates through two concurrent psychological processes, and that the translation of both into observable behavior varies with consumers’ need for self-expression. Two implications for theory follow, together with several for the management of a fan base.

Affective and identity-based routes have often been examined separately in research on consumer–brand relationships, leaving their empirical relationship to each other unspecified. The present results speak to it directly. Both predicted the two fandom behaviors strongly, yet correlated only weakly (r = 0.151). The two are therefore better treated as distinct psychological routes than as two intensities of one relationship. This does not establish that a consumer follows only one route, but a model containing either mediator alone would leave the other route unrepresented. The partially mediated model reported in Section 4.3 indicates that these are robust but not exhaustive pathways. Brand-related perceptions may therefore also reach fandom behaviors through processes the proposed model does not represent.

A second structural feature follows from the moderation results. Consumers’ expressive disposition operated on all four second-stage paths, so the two routes, distinct as they are, share the same expressive condition on the way to action. Brand fandom is therefore better described as a two-stage process with two distinct routes and a shared expressive condition than as a single bond of graded strength. Future research should test whether this structure holds for brands and categories in which innovation and community discourse are less central, and should examine how the two routes operate jointly within individual consumers.

These findings also offer important insights for high-tech brands’ fanbase management strategies. The two routes are better treated as separate design targets than as two ways of strengthening the same bond, and a brand that designs for only one of them will address only part of what drives its fan base. Initiatives that demonstrate the technological leaps, future vision, and direction of industry change the brand represents may be particularly suited to the affective route, rather than product promotion alone. New product launches, content showcasing the brand’s technological vision, and events and storytelling that embody the brand’s philosophy may encourage consumers to perceive the brand not merely as a product provider, but as a symbolic actor proposing a vision for the future. Direct experiential touchpoints that allow consumers to integrate the brand into their daily lives, tastes, and identities may be better suited to the identity route than increases in advertising exposure alone. Experience stores, onboarding services, user communities, and digital touchpoints that support usage routines can help strengthen the psychological connection between the brand and the consumer.

Furthermore, rather than managing fandom behaviors as a single engagement metric, brands need to distinguish between sharing and advocacy behaviors and creation behaviors. Sharing and advocacy behaviors are actions that spread brand meaning externally, such as recommendations, positive word-of-mouth, information sharing, and defending the brand or attempting to correct negative comments. To encourage these behaviors, brands must provide brand stories, product updates, comparative information, and community-oriented content that fans can easily explain and share. For creation behaviors, brands need to establish a framework that enables fans to turn their experiences into content, such as review programs, challenges, content templates, creator support programs, and user case studies. Both behaviors were significantly predicted by both mediators, so the case for managing them separately rests on the difference in what they produce rather than on a difference in what drives them: the key to managing a fandom lies in designing fans not merely as recipients or buyers, but as participants who both spread and reproduce the brand’s meaning.

The need for self-expression can also serve as an important criterion for segmenting and activating fan communities. Because the need for self-expression was treated here as a relatively stable individual difference rather than a campaign outcome, the practical step is to give consumers high on this disposition a stage: content contests, social media challenges, user story campaigns, and community leader programs. The route is narrower for consumers low on the disposition rather than closed: the conditional effect of both mediators on sharing and advocacy behaviors remains substantial at lower levels of the moderator (0.405 and 0.343). For these consumers, the appropriate instruments are those with low barriers to entry—short reviews, simple recommendations, voting, and participation in Q&A sessions—rather than public content creation. They are also the consumers a brand is most likely to overlook, since the same internal response is less likely to be externalized by them and engagement metrics built on observable participation may therefore under-record them. Within this sample, expressive disposition offered a more relevant basis for differentiation than country or ownership status. No structural path differed between the Korean and U.S. subsamples or between owners and non-owners (Section 4.4), so there is no evidence that these relationships require market-specific redesign.

### 6.2. Limitations and Future Research

This study presents the following limitations and suggests directions for future research. First, this study utilized Tesla—a single high-involvement, high-innovation brand—as its empirical context. While Tesla is a suitable case study in which active fandom is prominently observed, it is also a unique brand that combines strong symbolic innovation, technological discourse, and community culture. Therefore, further verification is needed to determine whether the research findings hold true for low-involvement products, service brands, brands that emphasize tradition and heritage, or brands with relatively low levels of innovation. Future research should compare and validate the triadic brand-related perception framework developed in this study across various industries and brand types.

Second, since this study is based on cross-sectional data collected at a single point in time, it has limitations in rigorously demonstrating the temporal precedence of causal relationships among variables. The process by which brand perception is transformed into advocacy and creation behaviors through self-transcendent emotion and self-brand connection is likely to accumulate over time. Future research needs to more rigorously examine the dynamic process of fandom formation through longitudinal designs, data collection at separate time points, or experimental designs.

## Figures and Tables

**Table 1:** Measurement items and sources.

Factors	Measurement Items	References
Brand Experience	- I find this brand interesting in a sensory way. - I engage in physical actions and behaviors when I use this brand. - This brand results in bodily experiences. - I engage in a lot of thinking when I encounter this brand. - This brand stimulates my curiosity and problem solving.	Brakus et al. (2009)
Perceived Product Value	- This product has consistent quality. - This product is well-made. - This product would help me feel socially accepted in a positive way. - This product would improve the way I am perceived.	Sweeney and Soutar (2001)
Brand Innovativeness	- This brand is innovative. - This brand introduces new concepts to the market. - This brand generates creative ideas and offerings. - This brand has the potential for continued innovative activity.	Shams et al. (2015)
Self-Transcendent Emotion	- I feel deeply moved by this brand. - This brand feels heartwarming to me. - I feel emotionally touched by this brand. - This brand feels extraordinary and inspiring to me. - This brand strongly captures my attention. - This brand makes me feel that I am in the presence of something greater than myself.	J. Kim et al. (2021); Zickfeld et al. (2019)
Self-Brand Connection	- This brand reflects who I am. - I can identify with this brand. - I feel a personal connection to this brand. - I use this brand to communicate who I am to other people. - I think this brand helps me become the type of person I want to be.	Escalas and Bettman (2005)
Need for Self-Expression	- I enjoy expressing my personal taste and individuality through the brands and products I use. - I place high importance on behaving in ways that reflect my identity and values. - I prefer consumption activities that show others who I am and what I stand for.	Adapted from Carroll and Ahuvia (2006)
Sharing and Advocacy Behaviors	- I post or share content related to this brand on my social media or other online platforms. - I talk about my personal experiences with this brand in conversations or messages with others. - I often pass along information or news about this brand to my friends and acquaintances, even when they do not ask. - I actively recommend this brand to my friends and relatives. - If someone asks for advice, I express positive opinions about this brand. - When I hear negative comments about this brand, I defend it or try to correct the information.	J. Kim et al. (2021); Fullerton (2005); Xie et al. (2017); Schau et al. (2009); Muñiz and O’Guinn (2001)
Creation Behaviors	- I have created simple content related to this brand (e.g., reviews, analyses, photos, or videos) and shared it on online communities or social media. - I have used this brand’s products beyond their basic purpose, adapting them to my own way or needs. - I have developed my own tips, settings, or usage routines to make better use of this brand’s products or services.	Nikolinakou et al. (2021); Schau et al. (2009)

Note: One sensory item (“This brand makes a strong impression on my visual sense or other senses”) was excluded due to low squared multiple correlation.

**Table 2:** Demographic information of survey participants (N = 635).

Variable	Category	Number	Ratio (%)
Country	Korea	313	49.3
	United States	322	50.7
Ownership	Tesla owner	315	49.6
	Non-owner (interested)	320	50.4
Tesla experience duration *	Less than 6 months	142	22.4
	6 months to less than 1 year	144	22.7
	1 to less than 3 years	252	39.7
	3 years or more	97	15.3
Gender	Male	605	95.3
	Female	30	4.7
Age	20s	88	13.9
	30s	232	36.5
	40s	182	28.7
	50s	108	17.0
	60s or older	25	3.9
Monthly Income	Less than $2500	30	4.7
	$2500 to less than $5000	77	12.1
	$5000 to less than $7000	130	20.5
	$7000 to less than $10,000	226	35.6
	$10,000 or more	172	27.1

Note: Percentages may not sum to 100.0% due to rounding. Income was assessed in local currency (KRW/USD); U.S. dollar ranges are displayed for consistency. * Tesla experience duration refers to the length of vehicle ownership for owners and the length of brand exploration for non-owner respondents.

**Table 3:** Results of reliability and convergent validity test.

Variables	Measurement Questions	Standardized Loading	Standard Error	t Value (*p*)	CR	AVE	Cronbach α
Brand Experience	BE2	0.662			0.849	0.530	0.848
	BE3	0.769	0.073	15.980 ***			
	BE4	0.742	0.071	15.573 ***			
	BE5	0.718	0.070	15.172 ***			
	BE6	0.744	0.071	15.595 ***			
Perceived Product Value	PPV1	0.814			0.942	0.801	0.940
	PPV2	0.922	0.037	29.245 ***			
	PPV3	0.931	0.037	29.652 ***			
	PPV4	0.909	0.038	28.583 ***			
Brand Innovativeness	BI1	0.781			0.925	0.756	0.923
	BI2	0.881	0.040	24.916 ***			
	BI3	0.918	0.040	26.234 ***			
	BI4	0.891	0.041	25.292 ***			
Self-Transcendent Emotion	STE1	0.723			0.946	0.746	0.947
	STE2	0.839	0.037	28.743 ***			
	STE3	0.911	0.052	23.074 ***			
	STE4	0.916	0.051	23.204 ***			
	STE5	0.896	0.051	22.696 ***			
	STE6	0.883	0.052	22.356 ***			
Self-Brand Connection	SBC1	0.649			0.915	0.687	0.917
	SBC2	0.801	0.049	22.354 ***			
	SBC3	0.881	0.064	18.648 ***			
	SBC4	0.901	0.065	18.941 ***			
	SBC5	0.883	0.065	18.679 ***			
Sharing and Advocacy Behaviors	SAB1	0.701			0.892	0.580	0.891
	SAB2	0.770	0.058	18.056 ***			
	SAB3	0.799	0.059	18.675 ***			
	SAB4	0.767	0.057	17.987 ***			
	SAB5	0.770	0.058	18.054 ***			
	SAB6	0.756	0.057	17.731 ***			
Creation Behaviors	CB1	0.842			0.916	0.785	0.912
	CB2	0.939	0.034	30.791 ***			
	CB3	0.874	0.035	28.027 ***			

Measurement model fit: χ^2^(df = 472) = 740.683, χ^2^/df = 1.569, RMR = 0.027, GFI = 0.932, AGFI = 0.919, NFI = 0.956, TLI = 0.982, CFI = 0.984, RMSEA = 0.030. Note: The unstandardized loading of the first indicator of each construct was fixed at 1 for scaling, so no standard error or t value is estimated for it. CR = composite reliability; AVE = average variance extracted; α = Cronbach’s alpha. *** *p* < 0.001.

**Table 4:** Correlation matrix and AVE.

Variables	AVE	BE	PPV	BI	STE	SBC	SAB	CB
Brand Experience (BE)	0.530	**0.728**						
Perceived Product Value (PPV)	0.801	0.188 ***	**0.895**					
Brand Innovativeness (BI)	0.756	0.284 ***	0.198 ***	**0.869**				
Self-Transcendent Emotion (STE)	0.746	0.210 ***	0.153 ***	0.291 ***	**0.864**			
Self-Brand Connection (SBC)	0.687	0.413 ***	0.314 ***	0.387 ***	0.151 ***	**0.829**		
Sharing and Advocacy Behaviors (SAB)	0.580	0.338 ***	0.339 ***	0.410 ***	0.595 ***	0.555 ***	**0.761**	
Creation Behaviors (CB)	0.785	0.347 ***	0.248 ***	0.397 ***	0.528 ***	0.472 ***	0.651 ***	**0.886**

Note: Diagonal elements (bold) = square roots of AVE (average variance extracted); off-diagonal elements = inter-construct correlations. *** *p* < 0.001.

**Table 5:** Measurement invariance across country and ownership groups.

Grouping	Model	χ^2^	df	CFI	RMSEA	SRMR	Δχ^2^ (Δdf)	*p*	ΔCFI
Korea vs. U.S.	Configural	1230.548	944	0.983	0.031	0.034	—	—	—
	Metric	1252.624	970	0.983	0.030	0.035	22.076 (26)	0.685	0.000
	Scalar	1283.908	996	0.982	0.030	0.036	31.284 (26)	0.218	−0.001
Owner vs. Non-owner	Configural	1199.690	944	0.984	0.029	0.034	—	—	—
	Metric	1223.783	970	0.984	0.029	0.036	24.093 (26)	0.571	0.000
	Scalar	1248.206	996	0.985	0.028	0.036	24.423 (26)	0.552	0.001

Note: Korea n = 313, United States n = 322; owners n = 315, non-owners n = 320. Δχ^2^, *p*, and ΔCFI compare each model with the preceding, less constrained model.

**Table 6:** Results of hypothesis tests.

	Hypothesis (Path)	β	*t*	*p*	Decision	*R* ^2^
H1	Brand Experience → Self-Transcendent Emotion	0.126	2.802	0.005 **	Supported	0.113
H2	Perceived Product Value → Self-Transcendent Emotion	0.087	2.122	0.034 *	Supported	
H3	Brand Innovativeness → Self-Transcendent Emotion	0.244	5.531	<0.001 ***	Supported	
H4	Brand Experience → Self-Brand Connection	0.302	6.634	<0.001 ***	Supported	0.301
H5	Perceived Product Value → Self-Brand Connection	0.213	5.421	<0.001 ***	Supported	
H6	Brand Innovativeness → Self-Brand Connection	0.268	6.404	<0.001 ***	Supported	
H7	Self-Transcendent Emotion → Sharing and Advocacy Behaviors	0.524	12.447	<0.001 ***	Supported	0.600
H8	Self-Transcendent Emotion → Creation Behaviors	0.471	11.973	<0.001 ***	Supported	0.462
H9	Self-Brand Connection → Sharing and Advocacy Behaviors	0.484	11.241	<0.001 ***	Supported	
H10	Self-Brand Connection → Creation Behaviors	0.413	10.339	<0.001 ***	Supported	

Note: β = standardized path coefficient. * *p* < 0.05; ** *p* < 0.01; *** *p* < 0.001.

**Table 7:** Results of mediation analysis.

Hypothesis (Path)	Indirect Effect	BootLLCI	BootULCI	*p*
BE → STE → Sharing and Advocacy	0.078	0.024	0.135	0.006
PPV → STE → Sharing and Advocacy	0.045	0.004	0.088	0.028
BI → STE → Sharing and Advocacy	0.136	0.087	0.192	<0.001
BE → STE → Creation	0.072	0.023	0.130	0.005
PPV → STE → Creation	0.041	0.004	0.081	0.026
BI → STE → Creation	0.126	0.080	0.180	<0.001
BE → SBC → Sharing and Advocacy	0.173	0.122	0.236	<0.001
PPV → SBC → Sharing and Advocacy	0.101	0.065	0.143	<0.001
BI → SBC → Sharing and Advocacy	0.138	0.096	0.188	<0.001
BE → SBC → Creation	0.152	0.105	0.206	<0.001
PPV → SBC → Creation	0.089	0.058	0.126	<0.001
BI → SBC → Creation	0.121	0.084	0.166	<0.001

Note: Indirect effects are unstandardized. Bootstrap 95% CI based on 5000 resamples; significance indicated when CI excludes zero.

**Table 8:** Results of moderating effects.

Dependent Variable	Predictor	*B*	*SE*	*t*	*p*	LLCI	ULCI
Sharing and Advocacy Behaviors	Constant	0.876	0.347	2.528	0.012	0.196	1.556
	Self-Transcendent Emotion (X)	0.220	0.116	1.898	0.058	−0.008	0.448
	Need for Self-Expression (W)	0.159	0.123	1.299	0.195	−0.082	0.400
	X × W	0.103	0.040	2.587	0.010	0.025	0.181
	F = 209.153 (*p* < 0.001), *R*^2^ = 0.595						
Sharing and Advocacy Behaviors	Constant	1.219	0.358	3.408	<0.001	0.516	1.921
	Self-Brand Connection (X)	0.065	0.119	0.548	0.584	−0.168	0.298
	Need for Self-Expression (W)	0.024	0.119	0.198	0.843	−0.210	0.257
	X × W	0.166	0.039	4.288	<0.001	0.090	0.242
	F = 223.736 (*p* < 0.001), *R*^2^ = 0.515						
Creation Behaviors	Constant	0.888	0.377	2.357	0.019	0.148	1.629
	Self-Transcendent Emotion (X)	0.102	0.126	0.805	0.421	−0.146	0.349
	Need for Self-Expression (W)	−0.016	0.134	−0.188	0.906	−0.278	0.247
	X × W	0.133	0.043	3.070	0.002	0.048	0.217
	F = 139.571 (*p* < 0.001), *R*^2^ = 0.399						
Creation Behaviors	Constant	0.422	0.399	1.058	0.291	−0.362	1.206
	Self-Brand Connection (X)	0.221	0.132	1.665	0.096	−0.040	0.481
	Need for Self-Expression (W)	0.160	0.133	1.203	0.229	−0.101	0.421
	X × W	0.091	0.043	2.116	0.035	0.007	0.176
	F = 129.805 (*p* < 0.001), *R*^2^ = 0.382						

Note: *B* = unstandardized regression coefficient; *SE* = standard error; LLCI/ULCI = lower/upper bounds of 95% CI. Analyses conducted using SPSS PROCESS macro v4.2, Model 1 (Hayes, 2022).

## Data Availability

The data presented in this study are available on request from the corresponding author. The data are not publicly available due to the privacy and confidentiality of the participants.

## Appendix A

Four of the eight constructs—brand experience, perceived product value, brand innovativeness, and self-brand connection—were measured with the published item wording and are therefore not listed here; for self-brand connection, the response format was changed from the 0–100 sliding scale used by Escalas and Bettman (2005) to the five-point Likert format used throughout this questionnaire. This appendix gives item-level detail for the remaining four scales, together with the translation procedure. Of the 18 items listed, 14 are wording-level adaptations of published measurement items, and four were developed for this study from a construct or community practice that the source describes but does not operationalize as a survey item. The rationale for the dimension-level decisions is given in Section 3.2. Items measuring brand experience and creation behaviors additionally carried context-specific anchoring examples in parentheses for the electric-vehicle setting (for example, for the behavioral brand-experience item: using features or modes, using the app, checking charging stations, operating the screen).

**Table A1:** Item-level adaptation for the four scales whose items were modified.

Source and Original Wording	Item Administered in This Study	Nature of the Change
“This brand reflects my personality”; “This brand is an extension of my inner self” (Carroll & Ahuvia, 2006, inner self)	I enjoy expressing my personal taste and individuality through the brands and products I use.	Adapted—referent shifted from a specific brand to the respondent’s general consumption disposition.
“This brand symbolizes the kind of person I really am inside” (Carroll & Ahuvia, 2006, inner self)	I place high importance on behaving in ways that reflect my identity and values.	Adapted—recast as the importance placed on identity-consistent action.
“This brand contributes to my image”; “This brand has a positive impact on what others think of me” (Carroll & Ahuvia, 2006, social self)	I prefer consumption activities that show others who I am and what I stand for.	Adapted—referent shifted to consumption activity; other-directed emphasis retained.
“I was moved” (Zickfeld et al., 2019)	I feel deeply moved by this brand.	Adapted—referent made specific to the brand.
“It was heartwarming” (Zickfeld et al., 2019)	This brand feels heartwarming to me.	Adapted—referent made specific to the brand.
“I was touched” (Zickfeld et al., 2019)	I feel emotionally touched by this brand.	Adapted—referent made specific to the brand.
“Feel special seeing it” (J. Kim et al., 2021, euphoria)	This brand feels extraordinary and inspiring to me.	Adapted—appraisal prompt written as a full statement.
“Stop and stare at it” (J. Kim et al., 2021, enthrallment)	This brand strongly captures my attention.	Adapted—appraisal prompt written as a full statement.
“Feel the presence of something greater than myself” (J. Kim et al., 2021, vastness)	This brand makes me feel that I am in the presence of something greater than myself.	Adapted—wording retained; stated as a response to the brand.
“Tweet about it, post on Facebook, or Instagram” (J. Kim et al., 2021, desire to save and share)	I post or share content related to this brand on my social media or other online platforms.	Adapted—named platforms generalized.
“Want to talk to my family, friends about it” (J. Kim et al., 2021, desire to save and share)	I talk about my personal experiences with this brand in conversations or messages with others.	Adapted—reworded around experience sharing; extended to written messages.
Customer citizenship behavior toward other customers (Xie et al., 2017)	I often pass along information or news about this brand to my friends and acquaintances, even when they do not ask.	Developed—unsolicited help to other customers, with brand information in place of service help.
“I will encourage friends and relatives to do business with [brand]” (Fullerton, 2005, advocacy intentions)	I actively recommend this brand to my friends and relatives.	Adapted—retail-service wording replaced by brand wording.
“I will recommend [brand] to someone who seeks my advice” (Fullerton, 2005, advocacy intentions)	If someone asks for advice, I express positive opinions about this brand.	Adapted—solicited character retained.
Evangelizing and justifying practices (Schau et al., 2009); oppositional loyalty (Muñiz & O’Guinn, 2001)	When I hear negative comments about this brand, I defend it or try to correct the information.	Developed—community practices stated as an individual behavior covering defense and correction.
“Write reviews or evaluations about a product or service”; “Write (initiate) posts (photo, video, posts or stories)” (Nikolinakou et al., 2021)	I have created simple content related to this brand (e.g., reviews, analyses, photos, or videos) and shared it on online communities or social media.	Adapted—creation and posting combined into one item.
Commoditizing practice (Schau et al., 2009)	I have used this brand’s products beyond their basic purpose, adapting them to my own way or needs.	Developed—use outside the marketed purpose.
Customizing practice (Schau et al., 2009)	I have developed my own tips, settings, or usage routines to make better use of this brand’s products or services.	Developed—modification of the product for personal use.

Note: Items are reported in their English administration form. “Adapted” marks an item modified from a published measurement item; “Developed” marks an item composed for this study from a construct or community practice that the source describes but does not operationalize as a survey item.

Because data were collected in Republic of Korea and the United States, the English items were translated into Korean and the two versions were compared item by item; discrepancies in meaning between the original and the translation were reviewed and adjusted before fielding. The measurement invariance tests reported in Section 4.1 provide empirical evidence that the two language versions function equivalently: full scalar invariance held across the Korean and U.S. subsamples, indicating that item loadings and intercepts are equivalent across the two country–language groups.

No expert panel review or pilot test was conducted prior to data collection. The adapted items were instead evaluated psychometrically after collection, and all constructs met the conventional thresholds for reliability, convergent validity, and discriminant validity reported in Section 4.1. The absence of formal pretesting is a limitation of this study.

## Appendix B

This appendix reports the alternative model specification referred to in Section 4.3. In the hypothesized model, the three brand-related perceptual antecedents are constrained to influence the two fandom behaviors only indirectly, through self-transcendent emotion and self-brand connection. To examine whether this constraint is empirically defensible, a partially mediated model was estimated in which six direct paths from the antecedents to the two behavioral outcomes were additionally freed. This model fits better than the hypothesized model (Δχ^2^(6) = 33.641, *p* < 0.001). Indirect effects were re-estimated with 5000 bootstrap resamples.

**Table A2:** Direct effects in the partially mediated model.

Path	β	z	*p*
Brand Experience → Sharing and Advocacy Behaviors	0.025	0.682	0.495
Perceived Product Value → Sharing and Advocacy Behaviors	0.117	3.644	<0.001 ***
Brand Innovativeness → Sharing and Advocacy Behaviors	0.082	2.375	0.018 *
Brand Experience → Creation Behaviors	0.087	2.224	0.026 *
Perceived Product Value → Creation Behaviors	0.045	1.306	0.192
Brand Innovativeness → Creation Behaviors	0.120	3.217	0.001 **

Note: β = standardized path coefficient. * *p* < 0.05; ** *p* < 0.01; *** *p* < 0.001.

**Table A3:** Indirect effects in the partially mediated model.

Path	Indirect Effect	BootLLCI	BootULCI	*p*
BE → STE → Sharing and Advocacy	0.072	0.021	0.127	0.008
PPV → STE → Sharing and Advocacy	0.039	0.001	0.078	0.048
BI → STE → Sharing and Advocacy	0.123	0.076	0.174	<0.001
BE → STE → Creation	0.064	0.019	0.114	0.009
PPV → STE → Creation	0.035	0.001	0.070	0.049
BI → STE → Creation	0.109	0.066	0.156	<0.001
BE → SBC → Sharing and Advocacy	0.142	0.097	0.197	<0.001
PPV → SBC → Sharing and Advocacy	0.081	0.049	0.115	<0.001
BI → SBC → Sharing and Advocacy	0.111	0.073	0.154	<0.001
BE → SBC → Creation	0.113	0.073	0.161	<0.001
PPV → SBC → Creation	0.064	0.037	0.095	<0.001
BI → SBC → Creation	0.088	0.056	0.127	<0.001

Note: Indirect effects are unstandardized. Bootstrap 95% CI based on 5000 resamples. BE = brand experience; PPV = perceived product value; BI = brand innovativeness; STE = self-transcendent emotion; SBC = self-brand connection.

As shown in Table A3, all twelve indirect effects remain significant when the direct paths are freely estimated, and their magnitudes are essentially unchanged relative to the hypothesized model. Four of the six direct paths reach significance; in the terms of Zhao et al. (2010), these are instances of complementary mediation, while the two that do not—brand experience to sharing and advocacy behaviors (β = 0.025, *p* = 0.495) and perceived product value to creation behaviors (β = 0.045, *p* = 0.192)—are instances of indirect-only mediation. Experiential perception therefore reaches sharing and advocacy behaviors only through the two mediators, as does value perception in the case of creation behaviors.

## Appendix C

This appendix reports the joint moderated mediation model referred to in Section 4.4. Both mediators, the need for self-expression, and the two second-stage interaction terms were entered simultaneously for each behavioral outcome. Indices of moderated mediation were obtained with 5000 bootstrap resamples. Predictor variables were mean-centered prior to forming the interaction terms.

**Table A4:** Index of moderated mediation for the twelve conditional indirect paths.

Conditional Indirect Path	Index	BootLLCI	BootULCI
BE → STE → Sharing and Advocacy	0.0099	0.0020	0.0211
PPV → STE → Sharing and Advocacy	0.0059	0.0003	0.0135
BI → STE → Sharing and Advocacy	0.0183	0.0056	0.0326
BE → SBC → Sharing and Advocacy	0.0425	0.0261	0.0614
PPV → SBC → Sharing and Advocacy	0.0283	0.0169	0.0422
BI → SBC → Sharing and Advocacy	0.0389	0.0242	0.0569
BE → STE → Creation	0.0149	0.0035	0.0298
PPV → STE → Creation	0.0089	0.0007	0.0193
BI → STE → Creation	0.0274	0.0101	0.0484
BE → SBC → Creation	0.0230	0.0067	0.0407
PPV → SBC → Creation	0.0153	0.0045	0.0278
BI → SBC → Creation	0.0211	0.0064	0.0372

Note: Bootstrap 95% CI based on 5000 resamples; an index is significant when the interval excludes zero. BE = brand experience; PPV = perceived product value; BI = brand innovativeness; STE = self-transcendent emotion; SBC = self-brand connection.

All twelve indices exclude zero, so the strength of every indirect pathway from brand-related perception to fandom behavior depends on the consumer’s need for self-expression. Consistent with the simple slope analyses in Figure 2, the second-stage coefficients rise across levels of the moderator: for sharing and advocacy behaviors, the self-brand connection path increases from 0.343 at one standard deviation below the mean to 0.653 at one standard deviation above, and for creation behaviors, the self-transcendent emotion path increases from 0.342 to 0.557 over the same range.
